# Supplementary figures and images for: 17R/S-Benzo-RvD1, a synthetic resolvin D1 analogue, attenuates neointimal hyperplasia in a rat model of acute vascular injury
Source: PLoS One. 2022 Feb 28;17(2):e0264217. doi: 10.1371/journal.pone.0264217 (PMC8884511; doi:10.1371/journal.pone.0264217)

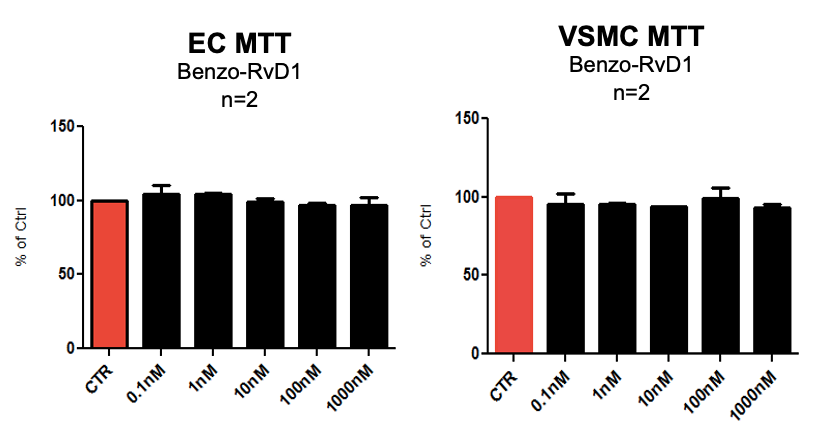

Supplement: S1 Fig — Benzo-RvD1 (0.1-1000nM) did not demonstrate significant cytotoxicity in endothelial cells (EC) and vascular smooth muscle cells (SMC) on a standard MTT assay. (TIF) [file pone.0264217.s001.tif]

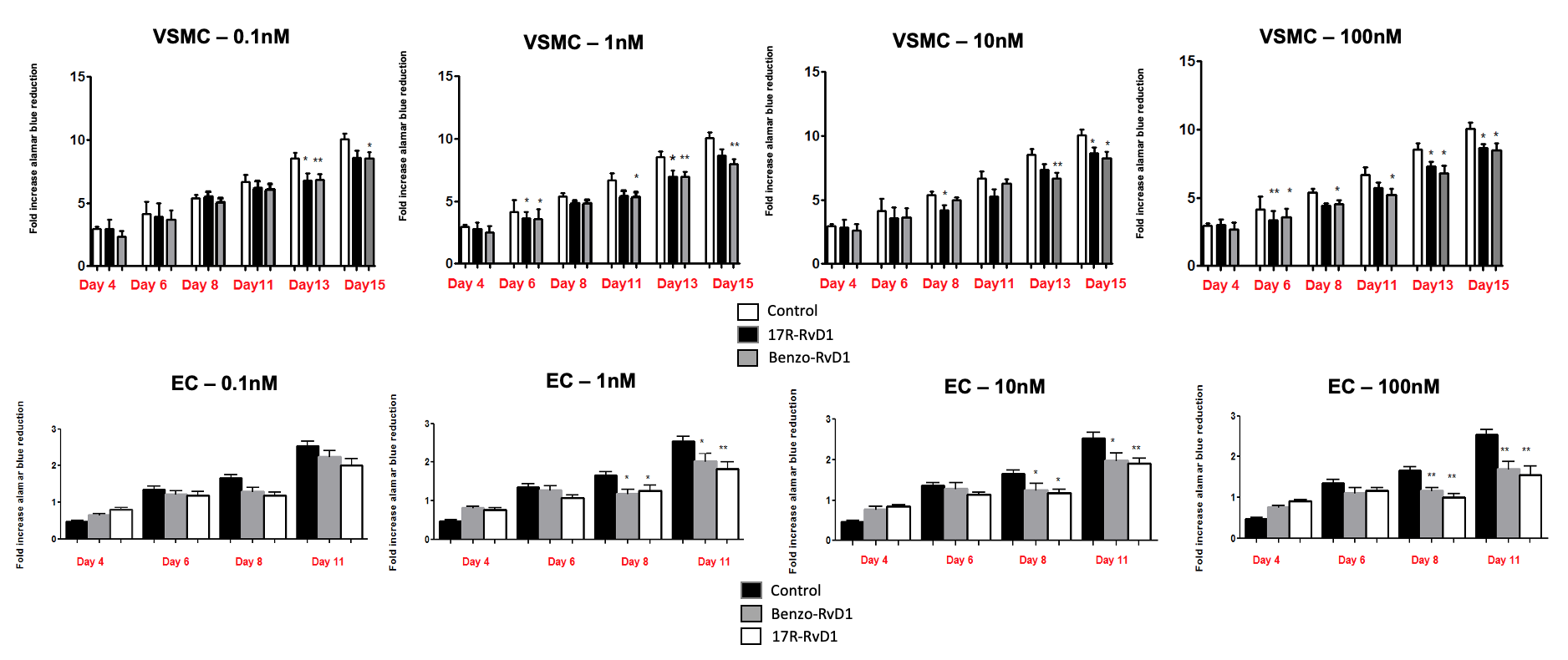

Supplement: S2 Fig — Treatment of VSMCs with Benzo-RvD1 and 17R-RvD1 leads to a significant, modest reduction in cellular proliferation across a wide range of concentrations and time points (*<0.05; **<0.001, n = 4 all timepoints). Treatment of ECs with Benzo-RvD1 and 17R-RvD1 leads to a significant, modest reduction in cellular proliferation only at later time points. (*<0.05; **<0.001, n = 4 all timepoints). Bars represent SEM. (TIF) [file pone.0264217.s002.tif]
