# Supplementary material for: 17R/S-Benzo-RvD1, a synthetic resolvin D1 analogue, attenuates neointimal hyperplasia in a rat model of acute vascular injury
Source: PLoS One. 2022 Feb 28;17(2):e0264217. doi: 10.1371/journal.pone.0264217 (PMC8884511; doi:10.1371/journal.pone.0264217)
Supplement: Attachment — Submitted filename: Response to Reviewers Updated v2.docx [file pone.0264217.s004.docx]

Michael Bader, Academic Editor

PLOS ONE

Re: PONE-D-21-24245

17R/S-Benzo-RvD1, a synthetic resolvin D1 analogue, attenuates neointimal hyperplasia in a rat model of acute vascular injury

Dear PLOS-One Reviewers,

Thank you for reviewing our submitted work. Below are our responses to the comments and questions from the review:

Responses to general guidelines

1. Edited manuscript to meet PLOS ONE’s style requirements, including the file name
2. Added (1) methods of sacrifice, (2) methods of anesthesia and analgesia
3. Included statement “This does not alter our adhered to PLOS ONE policies on sharing data and materials” and included updated Competing Interests statement on cover letter
4. Included ORCID iD for Michael S. Conte
5. Included supplemental figure data pertinent to the section that previously stated “data not shown” (toxicity) proliferation figure is now supplemental figure 2.

Responses to individual reviewer questions/comments

1. Add a reference for the two first sentences of the introduction
   a. Added references for the two first sentences of the introduction
2. In the statistical analysis paragraph of the Material and methods section, add information about the minimum p from which data were considered significant. For example, p<0.05.
   a. Added information regarding the minimum p from which data were considered significant, which was p<0.05
3. In multiple group analyses, one way ANOVA followed by Bonferroni or Tuckey post-hoc tests may better apply in this context. The authors can either repeat the appropriate statistics tests when applicable, or justify their choice in a limitation section.
   a. We performed ANOVA with post-hoc Tukey for individual group comparisons and edited the manuscript text to reflect this.
4. As the `n` are low, for transparency, the authors are invited to present all bar graphs as scatter/dot plot graphs, showing mean’s and SEM’s bars. (except graphs shown in supplementary figures, as scatter/dot plot may be less readable than bar graphs).
   a. All bar graphs (with exception of supplemental figures) were switched to scatter plots with means and SEM bars.
5. Figure 3 and 4, add a scale for the pictures.
   a. Figure 3 and 4 – added scale bars
6. Add a limitation section after the discussion and before the conclusion to address the main challenges met by this study, the limitations, and possible ways to overcome them in future investigations. This can include justification of the small number of samples n = 4 or 3; feasibility of further experiments or analyses (qPCR, western blot, etc).
   a. Added a limitation section following the discussion
7. Add a Highlight section with up to 5 bullet points to describe the main findings of the paper or/and patient-oriented potential applications.
   a. Highlight section added with 3 bullet points to describe the main findings of the paper

Thank you for considering our revised manuscript for publication. We look forward to any additional comments or questions.

Sincerely,

Michael S. Conte MD

Alexander Kim MD
